# Exploiting prior knowledge about biological macromolecules in cryo-EM structure determination

**DOI:** 10.1107/S2052252520014384

**Published:** 2021-01-01

**Authors:** Dari Kimanius, Gustav Zickert, Takanori Nakane, Jonas Adler, Sebastian Lunz, Carola-Bibiane Schönlieb, Ozan Öktem, Sjors H. W. Scheres

**Affiliations:** a MRC Laboratory of Molecular Biology, Cambridge, United Kingdom; b Department of Mathematics, Royal Institute of Technology (KTH), Sweden; c DeepMind, United Kingdom; d Department of Applied Mathematics and Theoretical Physics, University of Cambridge, Cambridge, United Kingdom

**Keywords:** 3D reconstruction, image processing, single-particle cryo-EM, imaging, structure determination, cryo-electron microscopy

## Abstract

Three-dimensional reconstruction of the electron-scattering potential of biological macromolecules from electron cryo-microscopy (cryo-EM) projection images is an ill-posed problem. The most popular cryo-EM software solutions to date rely on a regularization approach that is based on the prior assumption that the scattering potential varies smoothly over three-dimensional space. Although this approach has been hugely successful in recent years, the amount of prior knowledge that it exploits compares unfavorably with the knowledge about biological structures that has been accumulated over decades of research in structural biology. Here, a regularization framework for cryo-EM structure determination is presented that exploits prior knowledge about biological structures through a convolutional neural network that is trained on known macromolecular structures. This neural network is inserted into the iterative cryo-EM structure-determination process through an approach that is inspired by regularization by denoising. It is shown that the new regularization approach yields better reconstructions than the current state of the art for simulated data, and options to extend this work for application to experimental cryo-EM data are discussed.

## Abstract 2

The incorporation of prior knowledge about the structures of biological macromolecules into the reconstruction process of cryo-EM structure determination is proposed. Using a novel algorithm inspired by regularization by denoising, it is shown how convolutional neural networks can be used within this framework to improve reconstructions from simulated data.

## 1. Introduction   

In cryo-EM single-particle analysis, the three-dimensional structure of biological macromolecules is reconstructed from two-dimensional projection images of multiple copies of these molecules in different relative orientations. The requirement to image under low-dose conditions, in order to limit damage to the radiation-sensitive samples, leads to high levels of noise in cryo-EM images and the need to average over many images. Moreover, individual macromolecules, or particles, adopt unknown orientations in the sample and may adopt multiple conformations. The resulting large numbers of unknown variables render the optimimization problem ill-posed, which when combined with the high noise level in the data leads to a challenging reconstruction problem.

The most widely used approach to cryo-EM structure determination to date is based on the maximum-a-posteriori (MAP) estimator. This approach was introduced to the cryo-EM field through its implementation in the *RELION* program (Scheres, 2012*a* ▸,*b* ▸). Other software packages, such as *Cryo­SPARC* (Punjani *et al.*, 2017 ▸) and *THUNDER* (Hu *et al.*, 2018 ▸), have since built on the same approach. The MAP approach differs from common alternative approaches in two aspects. Firstly, it marginalizes over the unknown orientation (and conformational class) assignments of the particles, and secondly, it uses an explicit penalization term as a regularizer.

Whereas previously popular software packages typically aimed to find the best orientation and class for each particle, the marginalization approach rather uses a weighted average of all possible orientations. Marginalization was introduced to the cryo-EM field by Sigworth (1998 ▸). Using simulated data, he showed that marginalization over the orientations for 2D alignment against a single reference leads to reduced sensitivity to the choice of the starting reference and to the ability to align images with very low signal-to-noise ratios. Marginalization over class assignments was later found to be particularly useful for 2D (Scheres *et al.*, 2005 ▸) and 3D (Scheres *et al.*, 2007 ▸) classification of experimental cryo-EM images. Since its original conception in *Xmipp* (Scheres *et al.*, 2005 ▸), marginalization over class assignments has been adopted in many software packages, including *RELION* (Scheres, 2012*b* ▸), *THUNDER* (Hu *et al.*, 2018 ▸), *Frealign*/*CisTEM* (Grant *et al.*, 2018 ▸), Sphire (Moriya *et al.*, 2017 ▸) and *cryoSPARC* (Punjani *et al.*, 2017 ▸).

In general, regularization can be used to prevent overfitting when solving an ill-posed problem. Before the introduction of the MAP approach, cryo-EM software tools would prevent overfitting mostly through heuristic low-pass approaches and Wiener filter approaches (Penczek, 2010 ▸; Frank, 2008 ▸). The MAP approach pioneered the optimization of an explicitly regularized target function by expressing the reconstruction problem in an empirical Bayesian framework. The prior information that underlies the regularization in this approach is the assumption that rapidly changing density values in real-space cryo-EM reconstructions, or high powers at higher spatial frequencies in Fourier space, are unlikely. In other words, one assumes that cryo-EM reconstructions are smooth. By expressing both the (marginal) likelihood function and the prior in Fourier space, a standard *L*_2_ Tikhonov regularization approach is formulated (Engl *et al.*, 1996 ▸). Its MAP solution can be obtained through expectation–maximization (Dempster *et al.*, 1977 ▸), where an analytical solution to the maximization step exists. The resulting algorithm yields a 3D Wiener filter that removes high-resolution noise from the reconstruction, while estimating all necessary parameters from the data (Scheres, 2012*b* ▸). The ability to obtain efficiently filtered reconstructions without the need for user-tunable parameters probably played an important role in the rapid uptake of MAP optimization in the field (Fernandez-Leiro & Scheres, 2016 ▸).

Nevertheless, the smoothness prior seems an information-poor choice when compared with the knowledge about biological macromolecules that has been acquired through decades of structural biology research. For example, we know that the density for the macromolecules in cryo-EM reconstructions is surrounded by flat solvent density and that proteins are made up of amino-acid chains that fold into well defined secondary-structure elements such as α-helices and β-strands. Although a wealth of structural information exists, it has in practice been difficult to handcraft a formulation of a prior function that can be incorporated into the optimization algorithm that underlies cryo-EM structure determination. Despite this, recent work has shown that real-space filtering of maps performed iteratively throughout the reconstruction can help to mitigate the effect of noise on alignment and hence improve convergence and final map quality (Ramlaul *et al.*, 2019 ▸, 2020 ▸).

Machine learning based on deep neural networks, or deep learning in brief, can capture prior information and has recently seen tremendous success in a wide range of computer vision tasks (Krizhevsky *et al.*, 2012 ▸; Ren *et al.*, 2015 ▸; Ronneberger *et al.*, 2015 ▸). Convolutional neural networks have been shown to perform equally well or better than conventional state-of-the-art methods in inverse problems for several imaging modalities (Arridge *et al.*, 2019 ▸), including denoising (Zhang *et al.*, 2017 ▸; Jifara *et al.*, 2019 ▸; Gondara, 2016 ▸), computed tomography (Adler & Öktem, 2017 ▸, 2018*b* ▸) and magnetic resonance imaging (Hammernik *et al.*, 2018 ▸; Mardani *et al.*, 2018 ▸). Generally, this performance is attributed to the ability of deep neural networks to efficiently learn statistical correlations about both the noise and the signal through the examples in the training data set.

Regularization by denoising (RED) is an image-recovery framework that enables the plugin of various denoisers into a MAP optimization protocol (Romano *et al.*, 2017 ▸). This includes many denoiser classes, including general noise-reduction algorithms such as block-matching and 3D filtering (BM3D), more specialized denoising algorithms (Jonić *et al.*, 2016 ▸), and also deep learning-based denoisers such as denoising auto-encoders and U-nets.

The RED framework was originally centered around an explicit expression for a prior that incorporates the output of the denoiser. This framework requires that the denoiser function fulfills two conditions: local homogeneity and Jacobian symmetry. However, most state-of-the-art denoisers, including denoising convolutional neural networks, fail to exhibit these properties, in particular Jacobian symmetry. Despite this, RED has been shown to also perform well with these denoiser functions. Hence, the explicit prior approach cannot explain the behavior of this framework. In response to these issues, Reehorst and Schniter proposed a new framework, score-matching by denoising (SMD), which showed that RED achieves its performance by approximating the ‘score’ or the gradient of the prior distribution. This approach circumvents the requirement for an explicit prior expression and further does away with the abovementioned conditions on the denoiser function (Reehorst & Schniter, 2018 ▸).

Here, we propose a cryo-EM structure-determination procedure that is inspired by the RED protocol to integrate a convolutional neural network to express prior information. Cryo-EM reconstruction typically uses a vector-valued regularization parameter to model variations in signal-to-noise ratios across different spatial frequencies. The so-called ‘gold-standard’ recommendations in the field are to estimate these through the Fourier shell correlation (FSC) between independently obtained reconstructions from halves of the data set (Henderson *et al.*, 2012 ▸). This estimate typically varies greatly between data sets and throughout different refinement steps. Therefore, we expand RED to account for a vector-valued regularization parameter that varies throughout the reconstruction and present a theoretical framework, inspired by SMD, to show that this approach is valid in the case of more realistic Gaussian noise. We further present a simple heuristic to prevent any resolution overestimations that may arise owing to the new prior in the most difficult cases. We explore the measured performance of the denoiser in different resolution domains to determine the confidence in the new prior, which is subsequently used to weight between the new prior and the classical exponential prior. We call this approach confidence-weighted regularization by denoising (CW-RED).

Because we train our convolutional neural network on tens of thousands of cryo-EM reconstructions from simulated data and test it using data that are simulated in the same manner, this work does not yet represent a finalized solution to improve cryo-EM reconstruction from experimental data. Rather, it serves as a general proof of principle that learned priors can improve cryo-EM reconstruction. Future research directions, such as the exploration of alternative optimization algorithms and different strategies to design and train the neural networks, are discussed.

## 2. Theory   

### 2.1. Mathematical formalization of structure recovery   

We consider structure recovery in single-particle cryo-EM based on a Bayesian formalization of the problem. The starting point is after the particle-picking step where one extracts 2D projection images from the electron micrographs. Each of these shows a single macromolecular structure, or particle. Typically, following a classification step, images of the same macromolecular structure are selected for further refinement. We may here assume that these 2D projection images are approximately centered (with in-plane 2D translation) with respect to the corresponding 3D particle and that minor adjustments remain for an optimal translational alignment.

To formalize the above, let 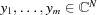 denote the discrete 2D Fourier transforms of the aforementioned 2D projection images. Likewise, let 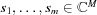 denote the discrete 3D Fourier transforms of the corresponding particles. This Fourier transform acts on the electrostatic potential generated by the particle and its surrounding aqueous buffer. Since all particles represent the same macromolecule up to an orientation and a translation, we have that *s_i_* = *t_i_* ○ *r_i_*(*x*), where 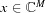 is the 3D Fourier transform of the molecule structure, *r_i_* ∈ *SO*(3) is its 3D rotation and *t_i_* ∈ *T*(3) is its 3D isomorphic real-space translation. We define the composite transformation *s_i_* := *t_i_* ○ *r_i_* and thus *g* ∈ *G* := *SE*(3) belongs to the special Euclidean group.

Structure recovery aims to reconstruct 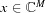 (the 3D Fourier transform of the structure of the molecule) from 2D data 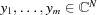 when the corresponding transformations *g*_1_, … *g_m_* ∈ *G* are unknown. It is natural to consider data as being generated by a random variable, *i.e.* there are 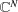-valued random variables 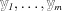 that generate the corresponding data. In the Bayesian setting one also adopts the viewpoint that the molecule structure and transformations are generated by random variables. Hence, we introduce a 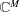-valued random variable 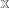 that generates molecular structures (models) and the *G*-valued random variable 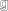 that generates particle transformations *g*_1_, … *g_m_*. Data 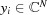 generated by the single particle 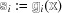 is then a single sample of the 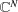-valued random variable 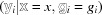, where
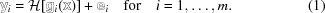
Here, 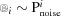 is a 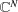-valued random variable representing noise in data and 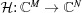 is the digitized (linear) model for TEM image formation in frequency space. In particular, 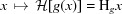 for some 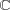-matrix 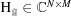 for fixed *g* ∈ *G*. The systematic effects of optical aberrations, such as the contrast transfer function (CTF), can be pre-calculated and given during refinement for each image. These effects can be incorporated into 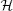, in which case 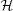 would be assigned a subscript *i* and considered unique for each particle image. However, to simplify the notation, this will be ignored in the following sections.

Assume next that the random variables 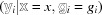 are independent and marginalize over the transformation 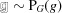 using a known (prior) probability density of orientations and translations in *G*. The joint probability density (joint data likelihood) for the entire data set 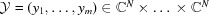 conditioned on the model 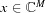 is then expressible as
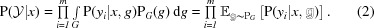
Note here that 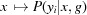 for fixed *g* ∈ *G* and 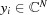 is given by (1), *i.e.* it is specified by the matrix H_*g*_ and the noise distribution 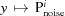.

The Bayesian approach to structure recovery aims to compute a suitable estimator that summarizes the (posterior) probability distribution of *x* given 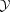. The density of this posterior distribution is expressible by Bayes’ theorem as
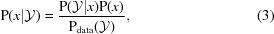
where P_data_ denotes the density for the joint distribution of 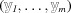.

### 2.2. MAP with a Gaussian prior   

The MAP estimator aims to find the model that maximizes the posterior probability (or equivalently its log-posterior probability), *i.e.* we seek a maximizer to 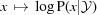. From (3) we obtain that a MAP estimator maximizes 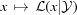, where
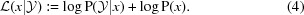
To proceed, we need to specify 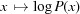 (the log-prior for 3D models) and 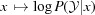 (the joint log–data likelihood).

Assume that data in frequency space is corrupted with additive uncorrelated Gaussian noise. More precisely, assume 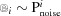 in (1) is a complex circularly symmetric Gaussian with mean zero and a diagonal covariance matrix with diagonal vector 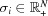 that encodes the frequency-dependent power of noise for each component of the *i*th image. Then,
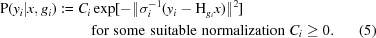
Next, most implementations of MAP-based cryo-EM structure recovery use an uncorrelated Gaussian prior for 3D models with zero mean. Stated more precisely, one assumes
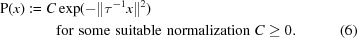
Here, 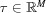 contains the frequency-dependent estimate of the power of the signal in the model. Such a distribution arises from applying the central limit theorem to 3D models with a random distribution of atoms, and can thus be intuitively regarded as a minimal assumption criteria about the 3D model (Wilson, 1949 ▸). More intuitively, this prior biases the recovery to a smoother real-space representation by suppressing large Fourier components.

A MAP estimator 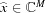 maximizes 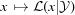, so in particular it solves 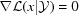. There is no closed-form solution for this equation, so one has to resort to iterative schemes. One example is the expectation–maximization (EM) algorithm (Dempster *et al.*, 1977 ▸).

For a data likelihood as in (5), the EM method generates iterates 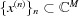 by computing 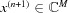 from data 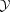 and the previous iterate 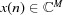. Given (4), (2) and (43) in Appendix *B* [App appb] it can be shown (Bendory *et al.*, 2020 ▸) that this is performed by solving the equation
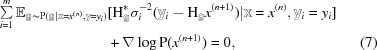
where H* is the adjoint of H with respect to the usual inner product on 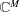 (see Appendix *B* [App appb]) and the expectation is weighted by
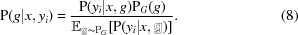
To simplify the notation in the following sections, we define
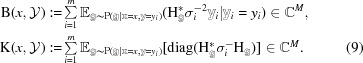
In the above, 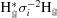 is diagonal owing to the Fourier slice theorem and the off-diagonal components are discarded.

Henceforth, when multiplying or dividing two vectors of equal length by each other, it is to be interpreted as applying these operations component-wise. This allows us to rewrite (7) as
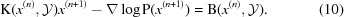
For a prior 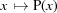 that is Gaussian as in (6), we obtain ∇logP(x) = −*r* ^−2^ *x*, so one can solve (7) (perform the M-step) analytically. This has a closed-form solution, generating the scheme
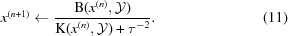
Additionally, we set 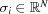 in (9) and the regularization parameter 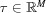. One common approach in line with the Bayesian viewpoint is to assume these are generated by random variables and then either marginalize over them or use estimates that are updated at every iteration *n*. As an example, the estimate of the regularization parameter τ is commonly based on the radially averaged Fourier shell correlation (FSC) calculated by comparing 3D models reconstructed from two independent halves of the data set (Scheres, 2012*b* ▸). More precisely, let 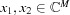 be two 3D models with associated data sets 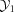 and 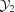, which are arrays (of equal length *m*/2) with elements in 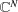. Next, define
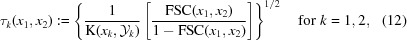
with FSC: 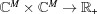 denoting the Fourier shell correlation (FSC). To use the above in (11), split the data into two sets 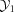 and 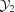. Let 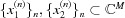 denote the two iterative sequences of 3D models obtained by applying the MAP EM scheme in (11) to 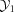 and 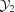, respectively. Instead of using a constant value for the regularization parameter τ, we instead use (12) to adaptively adjust its value based on these sequences, *i.e.* we replace the fixed 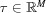 in (11) with 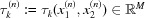 for *k* = 1, 2. Intuitively, this amounts to reducing the regularization applied in (11) when the FSC increases, which means that more signal is permitted to accumulate into each of the two reconstructions.

### 2.3. MAP with non-Gaussian prior   

Much of the usefulness of the EM method resides in the ability to perform the M-step in (7) in a computationally feasible manner. This is possible for a Gaussian prior and results in the EM scheme given in (11). Here, we consider priors 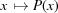 that are ‘close’ to a Gaussian in the sense that the second term below varies slowly with *x*:
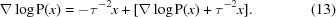
For this class of priors, (10) reads as
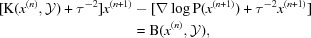
which has the following approximate solution:
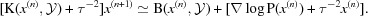
Using this approximation, we can compute an approximate MAP estimator using the scheme
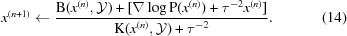
The above requires one to specify the ‘non-Gaussian’ part 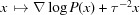. One can adaptively set τ as in the Gaussian case by splitting the data into two parts and using (12). Next, one may consider a data-driven approach to learn the ‘non-Gaussian’ part instead of handcrafting it.

The regularization by denoising (RED) method allows us to approximate the gradient of the log-prior using a neural network trained to denoise volumes. The RED prior formula has been shown to accurately estimate the gradient of the prior through score matching by denoising (SMD; Reehorst & Schniter, 2018 ▸). When dealing with independent additive Gaussian noise, RED can be used to integrate an external denoiser into an iterative image-restoration protocol (Romano *et al.*, 2017 ▸). We show in Appendix *A* [App appa] that the MMSE estimator 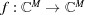 under Gaussian noise with covariance τ^−2^ approximates the gradient of the log-prior according to ∇log*P*(*x*) ≃ *f*(*x*) − τ^−2^(*x*). However, based on empirical observations, a more conservative approach is to also modulate *f*(*x*) with τ^−2^, which would suppress the influence of the denoiser when certainty in the data is high. Hence, we propose the gradient log-prior expression
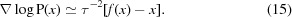
Inserting (15) into (14) gives an approximate M-step for priors using a learned denoiser:
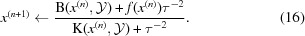
The input to the denoiser in this update formula is *x* ^(*n*)^, which lacks the information extracted in the latest E-step. However, both K and B can be evaluated at this stage, hence through them it is computationally feasible to provide the denoiser with the most ‘up-to-date’ information. Additionally, based on empirical observations we introduce a heuristic parameter, λ, to tune the influence of the denoiser in the M-step. Conclusively, we propose the following update scheme:
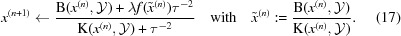
Note that (17) is equivalent to (11) when λ = 0 , so 0 ≤ λ ≤ 1 is an empirical parameter that balances the influence of denoiser versus conventional Gaussian prior. Furthermore, 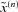 in (17) is equivalent to *x* ^(*n*+1)^ in (11) if τ^−2^ = 0. Hence, the denoiser acts on the unregularized map that contains information from the most recent alignment of the experimental data, which alleviates the assumptions made for (14).

Further adaptation can be obtained by making use of the fact that *RELION* is used for the refinement. To do this, we consider a mimimum mean-square estimator (MMSE) 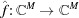 that is trained to ‘denoise’ *RELION* refinement volumes. More precisely, we consider the following supervised statistical learning problem:
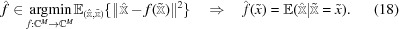
In the above, 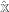 is the random variable that generates appropriately coarsened (low-pass filtered) 3D structures of macromolecular assemblies from the PDB and 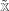 is the random variable that generates *RELION* refinement structures.

Since we lack knowledge about the joint probability distribution of 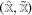, the conditional expectation in the left-hand side of (18) is replaced by its empirical counterpart given by supervised training data 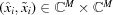 for *i* = 1, …, *n* that are random draws from 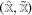, *i.e.* 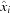 is the true 3D structure derived from the PDB (after appropriate coarsening) and 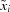 is a corresponding *RELION* refinement structure. Furthermore, a deep neural network is used to parameterize 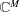-valued mappings on 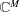, thereby replacing the infinite-dimensional minimization over such functions with a finite-dimensional minimization over the deep neural network parameters. The resulting counterpart of (18) can then be written as the following empirical risk-minimization problem:
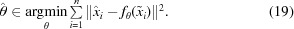
If the network has sufficient model capacity and there is sufficient supervised training data, then the above yields an approximation to the conditional mean estimator, *i.e.* 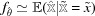.

## 3. Experimental design   

### 3.1. Convolutional neural network   

Synthetic training data were generated from 543 atomic structures that were downloaded from the Protein Data Bank (PDB; Berman *et al.*, 2000 ▸). All downloaded structures were solved using X-ray crystallography to at least 4 Å resolution, had molecular weights between 40 and 100 kDa, and consisted of a single protein chain; 110 structures also contained nucleic acids. Atomic models were converted to volumetric density maps in the MRC format (Crowther *et al.*, 1996 ▸) using the *pdb*2*map* tool in *EMAN*2 (Tang *et al.*, 2007 ▸). In order to obtain simulated projection data sets at different signal-to-noise ratios (SNRs), the intensity values of these maps were multiplied by three different constants: 0.014, 0.012 and 0.010.

Next, the density maps were projected onto images of 96 × 96 pixels, with a pixel size of 1.5 Å, and independent Gaussian noise of unity variance was added. The choice for a relatively large pixel size reduces computational costs, but was primarily motivated by the observation that SNRs in typical cryo-EM images at spatial frequencies higher than 3 Å are so low that they no longer effectively contribute to particle alignment. The resulting projection data sets had SNRs in the range 0.0003–0.0100 and on average 0.0020, which was calculated as the average per-pixel variance of the projection images divided by the variance of the added noise. Because *RELION* reconstructions are corrected for the effects of the CTF, no attempts were made to model the typical CTF of an electron microscope at this stage. Individual projection data sets of 10 000 projections each were generated for the different structures and SNRs using random projection directions that uniformly sampled the orientation space. Each of these projection data sets was then used for standard, unmasked ‘3D auto-refinement’ in *RELION* (Scheres, 2012*b* ▸), which was started from a 30 Å low-pass filtered version of the ground-truth map. The new external reconstruct functionality in *RELION*-3.1 (see below) was used to obtain unregularized half-maps at each of the intermediate iterations of all refinements. In total, this process yielded over 25 000 unmasked 3D density maps at different stages of refinement. Of these maps, 23% had a resolution better than 4 Å and less than 2% had a resolution of 10 Å or worse. This data set was subsequently augmented through size-preserving rotations.

To verify the validity of the underlying assumptions about Gaussian distribution of noise for the derivations in the theory section, we also trained a network using pairs of noise-free ground-truth and pure Gaussian noise-distorted maps. For this purpose, we used the estimated frequency-space distribution of noise calculated from the *RELION* intermediate reconstructions. During training, random Gaussian noise was generated in frequency space matching this profile. The noise was then convoluted with a real-space mask that dampened the noise levels in the solvent region. However, unless specified, the presented results regard a denoiser trained on *RELION* intermediate reconstructions. All networks were trained and evaluated on real-space maps. In practice, each map update involves an inverse Fourier transform of 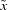 and a subsequent Fourier transform of the denoiser, *f*, before (17) is evaluated. To improve training convergence and robustness during inference, the input to the network was standardized through subtraction of the volume average and division by the volume standard deviation. The effect of the standardization was subsequently reversed via addition of the average and multiplication by the standard deviation of the original volume.

The presented approach extends the reconstruction protocol with a denoiser, which is a common image-to-image task with well established efficient network architectures. Hence, we limited the optimal architecture to hyperparameter optimization such as the depth and channel width of the network. For this work we chose a U-Net (Ronneberger *et al.*, 2015 ▸) for the denoiser, primarily motivated by the extensive research that has been performed on their application to denoising. Training and inference was performed using Python and Tensorflow 1.15 (Abadi *et al.*, 2015 ▸). At the start of this project, Tensorflow had the largest community and support compared with other available deep-learning frameworks, which we determined to be important for the accessibility of the implemented code and method to the scientific community. The network was trained with residual learning (Zhang *et al.*, 2017 ▸) and *L* ^2^ regularization of network weights via *Adam* (Kingma & Ba, 2014 ▸), with a mini-batch size of ten maps, for 27 epochs and with a constant learning rate of 10^−4^. The network was trained on an NVIDIA 2080 Ti graphics card until convergence for 21 h. The U-net consisted of five down-sampling and up-sampling stages that were implemented through average pooling and transposed convolutional layers (Dumoulin & Visin, 2016 ▸), respectively. Each stage was made up of two consecutive blocks repeating BN+ReLU+Conv. The kernel sizes of all convolutional layers were set to 3, while the number of channels for the first hidden layer was set to four. The number of channels was increased by a factor of two for each lower stage in the U-net. Experiments suggested that wider networks perform better when assessed with the mean squared error (MSE) to the ground truth. However, owing to memory limitations the above number of channels was chosen for the first input layer as the best balance between performance and memory requirements.

The input–output pairs for training consisted of the un­regularized and unmasked reconstructions from intermediate *RELION* iterations and their respective noise-free ground-truth maps. Here, the coarsening applied to the ground truth (see 19) was achieved by low-pass filtering it to match the resolution of the corresponding noisy input map. This was performed by multiplying the Fourier transform of the ground truth by the FSC of each noisy input map. The FSC was estimated for each map by *RELION* during the reconstruction of the training data set from the half-maps.

### 3.2. Assessment of the RED approach   

Four PDB structures were selected to test the performance of the RED approach (Table 1 ▸). The test structures were excluded from the training data set and had a minimum r.m.s.d. of 8 Å for at least 1000 aligned atom pairs with any other structure in the training data set. The four test structures represent different levels of difficulty for the reconstruction, mostly arising from differences in their molecular weight (MW) and their overall shapes (as projection images of near-spherical structures are harder to align than those with more anisotropic shapes). The test structures are listed in Table 1 ▸ in order of increasing difficulty, from the elongated, larger structure with PDB code 4ail to the smaller, near-spherically shaped structure with PDB code 4m82. Projection data sets for testing were made in the same way as the projection data sets that were used to generate the training set for the convolutional neural network. However, in contrast to the training data set, the test data set consists of images with a simulated CTF corresponding to a defocus range of 0.2μ to 2.0μ. We confirmed that using data with or without a CTF does not have any noticeable impact on the performance of the denoiser if changes in SNR are accounted for. This is expected, since the denoiser is only exposed to data where the effects of the CTF is mitigated through averaging over images with varying defocus. Test data sets were generated at four different SNRs by multiplying the original maps from the *pdb*2*map* tool by 0.022, 0.019, 0.016 and 0.013, which yields average SNRs of (i) 0.0038, (ii) 0.0028, (iii) 0.0019 and (iv) 0.0012, respectively.

The single-pass performance of the denoiser can be examined by applying it once to unregularized maps and evaluating the ratio between the *L* *^p^* difference to ground truth from the denoised map and the unregularized map. We evaluated the average of this ratio (using both *L* ^1^ and *L* ^2^) for the entire training data set as a function of the estimated nominal resolution of each map.

Standard, unmasked *RELION* 3D auto-refinements, with updates based on (11), were compared with refinements with updates based on (17). Again, all refinements were started from initial models that were obtained by 30 Å low-pass filtering of the ground-truth map and used the same refinement parameters. The results of both types of refinements were compared based on the reported half-map FSC and the FSC against the ground truth, and the angular error relative to the true projection angles. All maps were first multiplied with a solvent mask with a smooth edge before the FSC calculation.

### 3.3. Implementation details   

Instead of implementing the update formula in (17) directly in the C++ code of *RELION*, we created an external reconstruction functionality in its refinement program. When using the --external_reconstruct command-line option, the *relion_refine* program will write out MRC files containing B(*x*, *y*) and K(*x*, *y*) for both half-maps at each iteration step, together with the current estimate for FSC and τ^2^. The program then waits until reconstructions with a specified filename for both half-maps exist on the file system, reads those maps back in and proceeds with the next iteration of refinement. This functionality was coupled to a script in Python to perform the update formula in (17) using the pre-trained denoiser model. The Python script for external reconstruction using the proposed RED protocol, together with the architecture and the weights of the convolutional neural network that was used for the presented results, may be downloaded from github (https://github.com/3dem/externprior).

## 4. Results   

### 4.1. Performance of the denoiser   

**Figure 1:**
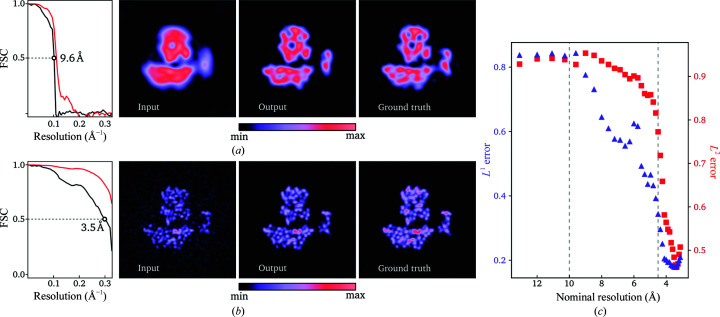
Single-pass denoising performance results at low (*a*) and high (*b*) resolution, showing central slices of the unregularized input map, the output from the denoiser and the ground truth for reconstructions of a structure from the test data set (PDB entry 4ail). The color scale spans between minimum and maximum density values in each slice. FSC curves show the correlation with the ground truth before (black) and after (red) denoising. The resolution at FSC = 0.5 is shown for each curve. (*c*) shows the average ratio between the *L^p^* difference from the ground truth to the denoised map and the unregularized map for the entire training data set as a function of the nominal resolution.

We first tested the effects of applying the denoiser to individual, intermediate, unregularized reconstructions from a standard *RELION* refinement [Figs. 1 ▸(*a*) and 1 ▸(*b*)]. After the first iteration of refinement of the PDB entry 4ail projection data at SNR (i), the unregularized half-reconstruction contains only lower resolution components and exhibits notable rotational smearing. The latter is often observed in low-resolution refinement intermediates and is caused by uncertainty in the angular assignments. Application of the denoiser reduces the rotational smearing and brings the map closer to the true signal, as confirmed by visual comparison and the FSC with the ground-truth map.

At iteration 16 of the same refinement, the unregularized map contains higher resolution components, but noise components are clearly visible in the volume surrounding the protein, which should be devoid of features. The denoiser efficiently removes the latter while retaining the same level of sharpness in the protein region and, again, both visual comparison as well as FSC calculation with the ground-truth map confirm that the denoised map is closer to the ground-truth signal than the unregularized map.

The average ratio between the difference to ground truth of the denoised map and the unregularized map was calculated for *L*_1_ and *L*_2_ at each nominal resolution [Fig. 1 ▸(*c*)]. When the resolution of the input map is worse than 10 Å the denoiser fails to produce a significant improvement on average. As the resolution improves beyond 10 Å the performance gradually improves and eventually plateaus beyond 4.5 Å.

### 4.2. Performance of the RED approach   

**Figure 2:**
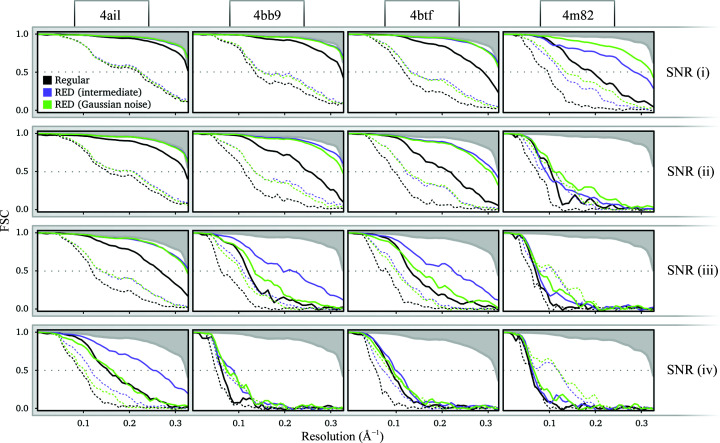
FSCs of reconstructions at four different SNRs (rows) of four structures (columns). Regular *RELION* (black) is compared with RED using a denoiser trained on Gaussian noise (green) and on *RELION* intermediate reconstructions (purple). Dotted lines show the half-map FSC and solid lines show the FSC between the regularized map and the ground truth. All maps were first multiplied with a solvent mask with a smooth edge before comparison with the ground truth. The upper shaded area shows the FSC when the model is reconstructed with zero angular error.

Next, we tested the performance of the RED approach on projection data sets of the four test structures at four different SNRs. We first ran the RED approach with a denoiser trained on Gaussian noise and compared the results with those for a denoiser trained on *RELION* intermediate reconstructions (Fig. 2 ▸). The RED-based approach outperforms the standard approach in all refinements performed, as measured by the FSCs between the resulting reconstructions and the ground-truth maps (solid green and purple lines in Fig. 2 ▸). This suggests that Gaussian noise can partially explain the distortions. However, the denoiser trained on *RELION* intermediate reconstructions performs better in most cases, confirming that Gaussian noise is an incomplete model of the distortions observed in *RELION* intermediate reconstructions.

Both the RED approach and standard *RELION* auto-refinement produced a reconstruction with a resolution close to the Nyquist frequency for the easiest test structure, PDB entry 4ail, at the highest SNR. Conversely, both approaches yielded a low-resolution solution that was devoid of recognizable protein features for the most difficult test structure, PDB entry 4m82, at the lowest SNR. Therefore, the range of structures and SNRs of our test data set represents a wide range of scenarios from easy to hard refinements. The gains in performance tend to be higher at higher SNRs; they are small for the most difficult refinements. It is noteworthy that in the most simple cases the RED approach achieves an FSC that is equal or slightly higher than that of the reconstruction with the true angles.

**Figure 3:**
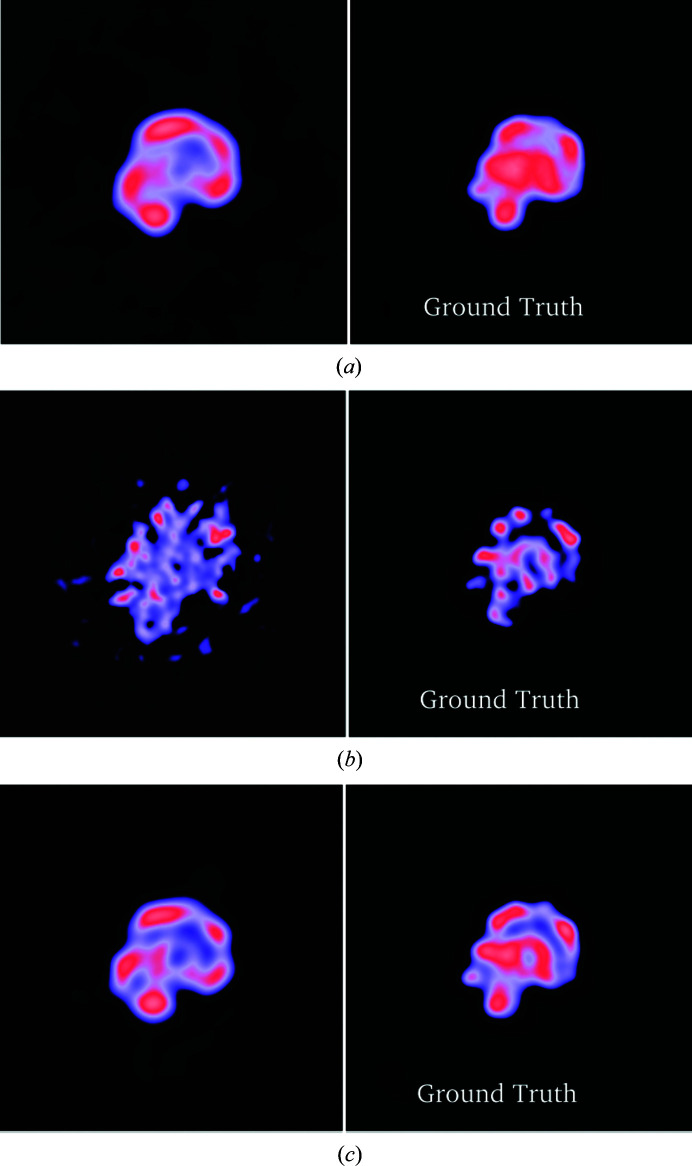
Central slices of the reconstruction results of PDB entry 4m82 at SNR (iv) and their respective ground truth. (*a*) Regular *RELION*, (*b*) RED, (*c*) confidence-weighted RED. Each ground-truth map has been low-pass filtered to match the estimated resolution of the reconstructed maps by multiplying their Fourier transform with the half-map FSC. The result for RED shows high-contrast features that do not correlate well with the ground truth. This issue is significantly alleviated by confidence weighting.

Measures of reconstruction quality that are reported by *RELION* without having access to the ground truth, *i.e.* the half-map FSCs, also improve when using the RED approach (dotted green and purple lines in Fig. 2 ▸). However, for the refinement of PDB entry 4m82 at the three lowest SNRs, we noticed a severe overestimation of resolution based on the half-map FSCs using the RED approach. A visual examination of the maps [Fig. 3 ▸(*b*)] reveals high-contrast features that are similar in appearance to those of biological macromolecules but do not correspond to the ground truth. This points to the denoiser introducing false high-resolution features into the reconstructions that are shared among the two half-maps. This then leads to an overestimation of the resolution, which is based on the correlation between the half-maps. Since the half-map FSC is the only reliable estimate of resolution when the ground truth is missing, this issue poses a major problem for the validation of the reconstruction results.

### 4.3. Confidence weighting   

The λ parameter in (17) can be used to fall back on the Gaussian prior in a scenario where confidence in the performance of the denoiser is lacking. Inspired by the results of the single-pass performance test of the denoiser [Fig. 1 ▸(*c*)], we tested a simple tuning scheme for λ to address the issue of resolution overestimation. We will refer to this modification as confidence-weighted RED (CW-RED).

At nominal resolutions worse than 10 Å, as estimated by *RELION*’s half-map FSC, we assign a full fall-back onto the Gaussian prior by setting λ = 0. This is where the single-pass performance indicates that the denoiser results in little improvement in the reconstruction [see Fig. 1 ▸(*c*)]. To avoid impairing the performance at high resolutions, however, we set λ = 1 at nominal resolutions better than 4.5 Å, which is approximately where the single-pass performance of the network begins to peak. For any nominal resolution between 4.5 and 10 Å we use a linear interpolation between 0 and 1.

**Figure 4:**
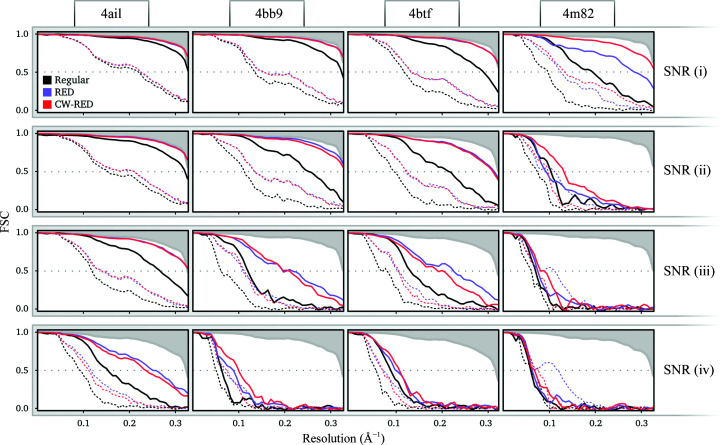
FSCs of reconstructions at four different SNRs (rows) of four structures (columns). Regular *RELION* (black) is compared with RED (purple) and confidence-weighted RED (red), in both cases using a denoiser trained on *RELION* intermediate reconstructions. Dotted lines show the half-map FSC and solid lines show the FSC between the regularized map and the ground truth. All maps were multiplied with a solvent mask with a smooth edge before comparison with the ground truth. The upper shaded area shows the FSC when the model is reconstructed with zero angular error. Note that the purple lines show the same results as the purple lines in Fig. 2 ▸.

**Figure 5:**
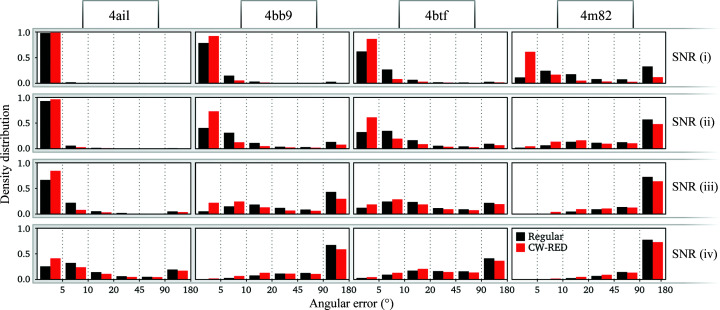
The distribution of angular error of reconstructions at four different SNRs (rows) of four structures (columns). Regular *RELION* (black) is compared with confidence-weighted RED (red). The error is defined as the axis-angle representation difference between the known rotation and the refined angle.

**Figure 6:**
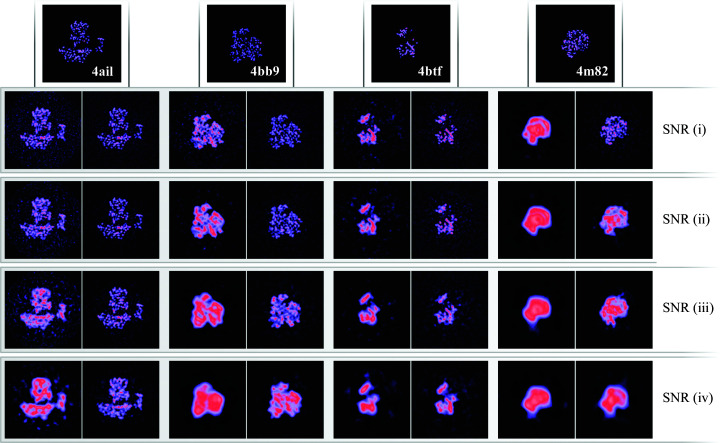
Central slices of the reconstructed maps in the test data set for the four different SNRs. Each pair compares regular *RELION* (left) with confidence-weighted RED (right). The top row shows the ground truth for the maps in each column.

Using this approach, we see that the overestimation of the half-map FSC is significantly alleviated (dotted red lines in Fig. 4 ▸) without affecting the overall quality of the reconstructions as measured by the FSC with the ground truth (solid red lines in Fig. 4 ▸), the errors in the angular assignments (Fig. 5 ▸) and the visual appearance of the resulting structures (Figs. 6 ▸ and 7 ▸). A visual inspection of the map for PDB entry 4m82 resulting from the refinement with the lowest SNR also suggests that the reconstruction no longer accumulates false structural features with poor correlation to the ground truth (Fig. 3 ▸).

## 5. Discussion   

Deep learning faces four well known general challenges: domain shift, brittleness, explainability and fairness. Domain shift can prevent the deep neural network (DNN) from generalizing beyond the training domain owing to shifts in distribution between the training data set and the data encountered when the DNN is deployed; brittleness is the sensitivity to small changes in the data; explainability refers to transparency in the features learned and the degree that they can be made subject to human interpretability; and fairness reflects insensitivity to input variables that should be statistically independent from the output. The manner in which the DNN is incorporated into the reconstruction process, proposed here, intentionally retains most of the established statistical model that has been handcrafted from physical principles, based on the image-formation process. This approach alleviates many of the concerns related to brittleness and explainability by minimizing the role of the DNN. Furthermore, the data used to train the DNN will in principle introduce bias. However, this would in principle hold for any choice of priors. Generally, a handcrafted prior is more biased than a trained one (Adler & Öktem, 2018*a* ▸). Therefore, fairness is of minor concern. However, domain shift is a considerable issue that will be further discussed in this section.

Convolutional neural networks for denoising are typically trained on pairs of noiseless and noisy representations of the signal. It is thus crucial to have access to an accurate noiseless ground-truth signal, which makes it challenging to apply these networks in areas where such a ground truth is impossible or expensive to acquire, such as medical imaging (Huo *et al.*, 2018 ▸). To circumvent this problem, we used synthetic ground-truth cryo-EM reconstructions and used a simplistic physical forward model to generate simulated projection images. In doing so, we explicitly did not aim to obtain a network to denoise reconstructions from experimental cryo-EM data, and thereby avoided some of the challenges of domain shift. Rather, we used test data sets that were generated using the same forward model to provide a proof of principle that learned priors can improve the reconstruction process. Our results convincingly make this case: the RED approach outperformed standard auto-refinement in *RELION* in all of the tests performed.

The results shown in Fig. 1 ▸ suggest that the denoiser has learned to differentiate protein from solvent regions and apply adequate amounts of filtering suited for each region, which is difficult to achieve using classical denoising algorithms. Additionally, in the easiest test cases the RED approach achieves an equal or higher FSC than the reconstruction using the true angles, which supports the idea that structural information can be injected into the reconstruction process through the proposed method.

The standard approach in *RELION* uses an *L*_2_ Tikhonov regularization on the Fourier components of the reconstruction (Scheres, 2012*b* ▸). In practice, this purely Fourier-based regularization term is often complemented with an *ad hoc* regularization in real space by setting all densities to a constant value in the volume outside a user-specified 3D mask around the reconstruction. Often, such masks are generated after an initial refinement has yielded a preliminary reconstruction. In the tests performed here, no solvent mask was provided. Thereby, the improvements observed using the RED approach reflect the difference between a purely Fourier-based *L*_2_ Thikonov regularization and a learned prior in real space. The observed differences with masked refinements would be smaller.

Although, aware of problems with domain shift, we used different macromolecular structures (*i.e.* with >8 Å r.m.s.d. on the atomic coordinates) in the test and training data sets, we still identified a risk of injecting similar, protein-like features into both half-maps, which then lead an overestimation of resolution based on the half-map FSC. Without access to the ground truth, the half-map FSC is the single most important measure of quality for the reconstruction, resulting in a major concern for the usefulness of the method. We observed that for our network this problem was most noticeable for reconstructions at resolutions lower than 10 Å, for which there were few examples in the training data set. More data in this domain will probably alleviate the problem. Still, we were able to address this issue with an empirical approach that we call confidence-weighted RED. By falling back onto the less informative Gaussian prior at nominal resolutions where the denoiser is known to produce little improvement in the reconstruction, we managed to sufficiently alleviate the problem while retaining equal overall performance. This approach is based on performance statistics gathered by applying the denoiser to the training data set, and required no significant additional effort compared with regular RED. To avoid the risk of overestimating the confidence in the denoiser, ideally another data set should be used instead of the training data set. It is also noteworthy that the performance of the denoiser is likely to vary over different resolution shells and that a local patch-based approach might also be required to better handle variations in local resolution. Therefore, a more detailed investigation into the confidence-weighting scheme could lead to further improvements.

We envision multiple other avenues for improving the approach set out in this paper. Firstly, different network architectures may be devised and networks may be trained through alternative loss functions to improve their performance. The macromolecular structures used for our training and test data sets were limited to contain only a single protein chain. Wider neural networks with more model capacity may be required to achieve comparable performance on experimental cryo-EM data. A large variety of successful network architectures have been developed in other areas of image processing, for example FFDNet (Zhang, Zuo *et al.*, 2018 ▸) and RCAN (Zhang, Li *et al.*, 2018 ▸). Furthermore, to handle larger reconstructions the denoiser may be trained on patches, rather than on the entire map as performed here, and the patches may be combined using a sliding window, similar to previous work in tomography (Tegunov *et al.*, 2020 ▸). This approach is appealing owing to the inherent flexibility in terms of particle/box size and memory requirements. Moreover, the use of patches may be intrinsically better suited to deal with the inherent variability in map quality and local resolution that is caused by the different extents of order that exist in many biological macromolecules. For instance, confidence weighting can easily be made to rely on the average local resolution in each patch rather than the entire volume. However, as networks trained on patches might no longer see the entire box, where a particle at the center is surrounded by solvent this approach may be less powerful in flattening the solvent. One solution could be the combination of multiple denoisers that are trained in different resolution domains. For instance, a patch-based denoiser dedicated to the high-resolution domain could be combined with a denosier with a global view of the map at low resolution. The confidence-weighting scheme introduced in this paper is well suited to combine each of these denoisers according to the performance in each of the resolution domains.

Secondly, to limit problems with domain shift, the networks should be optimized for the denoising of reconstructions from experimental cryo-EM data. Experimental cryo-EM data have different characteristics in both the signal and the noise to the synthetic data used here. Experimental noise is not independent in the pixels, but falls off with higher spatial frequencies. Both the signal and part of the noise are affected by the CTF of the microscope, and in particular the lowest spatial frequencies in experimental cryo-EM reconstructions are poorly modeled by the simplistic forward model of protein atoms in vacuum used in this paper. Several options exist to generate more realistic pairs of maps for training the denoiser. Refinements with experimental cryo-EM data downloaded from the EMPIAR database (Iudin *et al.*, 2016 ▸) may be used to generate training data. In such a scenario, generating image pairs with ground truth will be difficult, but one could generate intermediate reconstructions from relatively small subsets of the data and provide the high-resolution maps from the complete data sets as substitutes for the ground truth. This is particularly feasible when the networks will only be trained on intermediate-resolution reconstructions (also see below). Alternatively, one could train generative adversarial networks (GANs) to learn a data-driven forward model from pairs of atomic models and their corresponding noise-free cryo-EM reconstructions in order to generate more realistic ground-truth maps of disordered regions, for example membrane patches. For this purpose, conditional GANs (cGANs) are a particularly suitable candidate, since pairs of atomic model (ground truth) and reconstruction density are available (Isola *et al.*, 2017 ▸). Similarly, cycle-consistent adversarial networks (CycleGANs; Zhu *et al.*, 2017 ▸) or style-transfer GANs (Gatys *et al.*, 2016 ▸) may be used to relate the two data domains in cases where pairs are lacking, for example for low-resolution reconstructions without a matching ground truth or for generalizing the data set to new experimental conditions with few existing examples (Januszewski & Jain, 2019 ▸).

Thirdly, the approach described here may be adapted to use optimization algorithms other than expectation–maximization. A gradient-driven approach with mini-batches would be based on 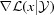 and (15) to inject prior knowledge into the reconstruction more often, which has the potential to improve the convergence speed. For this class of algorithms, adversarial regularisers might be a more natural candidate compared with RED, since this method models the prior more directly and thus enables better control of the properties of the generated gradient (Lunz *et al.*, 2018 ▸). Alternatively, algorithms based on ADMM and plug-and-play denoisers are of potential interest for investigation (Venkatakrishnan *et al.*, 2013 ▸; Bigdeli *et al.*, 2019 ▸).

Finally, an important consideration when employing prior information in solving any inverse problem is that the prior information used in solving the problem can no longer be used for external validation of the results. This touches on the challenge of explainability and is relevant when injecting prior information about biological macromolecules into the cryo-EM reconstruction process. In current cryo-EM approaches such information is not used at all, and the appearance of protein and nucleic acid-like features in cryo-EM reconstructions is often implicitly used to confer confidence in the correctness of the maps. However, one could imagine a more informative prior generating reconstructions in which such features are incorrectly ‘invented’ from noise in the maps. Commonly used procedures to prevent overfitting, most notably splitting the data set into two independent halves, do not necessarily protect against the interpretation of such false features. Therefore, new validation tools may need to be devised to guard against the interpretation of false features. One option could be to only use information up to a given intermediate resolution when training the denoiser. In this way, the presence of protein- or nucleic acid-like features beyond this resolution could still be used to provide confidence in the result. This approach is particularly appealing because the high-resolution signal typically does not contribute much to the alignment of the individual noisy projection images anyway (Henderson *et al.*, 2011 ▸).

We expect that with further improvements along the lines discussed above, the approach presented in this paper will result in a tool for structure determination that will also outperform the current state of the art for experimental cryo-EM data. This has the exciting potential to improve any reconstruction from existing data sets and to expand the applicability of the technique to samples that are currently beyond its scope.

## Figures and Tables

**Figure 7:**
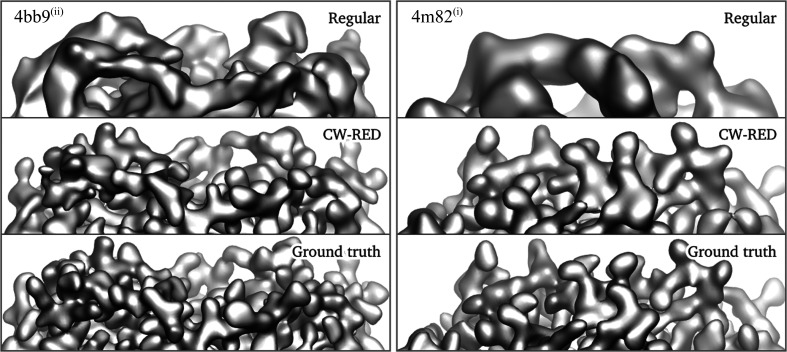
Isosurface visualizations of the reconstruction results with regular *RELION* and confidence-weighted RED together with the ground truth for PDB entries 4bb9 at SNR (ii) (left) and 4m82 at SNR (i) (right).

**Table 1:** Characteristics of structures in the test data set Compactness is expressed as the ratio between the smallest and the largest diameter of the molecule. Relative image SNR is expressed as the per-pixel average variance of signal over noise relative to that of the structure with the maximum SNR (PDB entry 4ail).

PDB code	4ail	4bb9	4btf	4m82
Molecular weight (kDa)	96	70	54	46
Relative image SNR	1.00	0.52	0.27	0.26
Compactness	0.42	0.64	0.62	0.74
α-Helical content (%)	38	49	46	39
β-Strand content (%)	21	10	10	12
Loop content (%)	36	41	44	49
Nucleotide content (%)	5	0	0	0

## Appendix A Dependent Gaussian noise

SMD describes the results of RED using a white Gaussian noise model with a fixed variance as the underlying assumption for the image distortion. This appendix describes details of how the RED approach was expanded to handle the type of distortions observed in cryo-EM data. In particular, we derive a dynamically adjusted regularization parameter, as described in (15), and a modified training scheme by managing the significantly varying levels of distortion in the data. We show how this leads to a training protocol that minimizes the model mapping to a coarsened ground truth. The aim of this appendix is not to provide a full mathematical proof of (15), but rather to present an approach that highlights the assumptions and approximations that are required to derive it.

We define the random vector , , where *P* here is the distribution of undistorted ground-truth data. Here, we assume a Gaussian noise model formalized as , where  and  is a covariance matrix that depends on  to describe the shape of the Gaussian. For the application in this paper, this dependency does not have to be explicitly defined; rather, an empirical approach is sufficient where the training data set is extended to also include observations of ν. In Section 3.1 we specify how this was performed in practice. One can device an empirical prior distribution that approximates *P* through kernel density estimation (KDE; Parzen, 1962 ▸), given a large representative corpus of training data, {*x*}*^T^*_*t*=1_, drawn from *P*, and the corresponding {*ν*}*^T^*_*t*=1_, as

For the continuum limit of KDE, we instead obtain

The gradient of the log-KDE-prior then becomes (see Appendix *B* [App appb] for the precise definition of ∇)

We define an MMSE function as

In practice, owing to the higher relative error of high-frequency components,  corresponds to a coarsened (low-pass filtered) version of . We also define the MMSE of  as

It then holds that

Now, it follows that

In the above, (31) uses the orthogonality principle and (33) follows from (28). In the particular case of *f*, in the above, belonging to a class of universal approximator functions, *e.g.* CNN denoisers, the minimization (*i.e.* training) is carried out over the network parameters θ with pairs of . It thus follows that optimizing the weights θ of such a function, , through

equates matching the gradient of the log-KDE-prior through

In this paper, we assume that *p*_KDE_ is a good enough approximation of *P* given the training-data size and data dimensionality. Conclusively, *f*_θ_ is trained to map a noisy data point, , to a weighted combination of low-pass filtered ground truths. We are, however, still required to estimate ψ. Ignoring the off-diagonal components of ψ and estimating only the diagonal components through (12), *i.e.* ψ ≃ τ^−2^, implies that (36) is equivalent to (15).

## Appendix B Wirtinger derivatives

To derive (7) one has to differentiate the expression for the posterior probability in (3). In this paper, it is expressed in the Fourier domain as a real function with complex input. Here, we define the gradient operator acting on such a function using the formalism of Wirtinger derivatives (see, for example, Appendix A of Fischer, 2005 ▸). More specifically, the Wirtinger derivatives of *f* with respect to the complex variables *z*_*i*_ = *x*_*i*_ + *iy*_*i*_ and  := *x*_*i*_ − *iy*_*i*_ are defined via

The corresponding gradients can then be defined as

We also use the shorthand notation . Note that  is stationary if and only if  or . It follows from Appendix A in Fischer (2005 ▸) that for  and

where *A** is the adjoint of *A* with respect to the usual inner product on . Hence, if we take  and *b* = σ_*i*_^−1^ *y_i_* we find

Similar calculations give the gradient of the Gaussian log-prior:

In this paper, to simplify notation outside this appendix, the subscript on ∇ is dropped when the differentiation is with respect to *x* or any variations thereof.
